# Meteorological Register

**Published:** 1814-10

**Authors:** C. Blunt

**Affiliations:** Philosophical Instrument Maker, No. 38, Tavistock-Street, Covent-Garden


### METEOROLOGICAL REGISTER.

*From August the 25th, to September the 25th,* 1814.

Kept by C. BLUNT, Philosophical Instrument Maker, No. 38, Tavistock-Street, Covent-Garden.

| Moon. | Day. | Wind. | Barometrical Pressure. Max. | Min. | Mean. | Temperature. Max. | Min. | Mean. | |
|---|---|---|---|---|---|---|---|---|---|
| | 26 | W | 29·86 | 29·73 | 29·805 | 72 | 57 | 62·6 | Fair |
| | 27 | WbyN | 29·98 | 29·91 | 29·945 | 71 | 58 | 62·6 | Fair |
| | 28 | W | 30·08 | 30·— | 30·04 | 70 | 56 | 61·6 | Fair |
| | 29 | NW | 30·16 | 30·11 | 30·145 | 69 | 55 | 60·6 | Fair |
| ○ | 30 | NW | 30·17 | 30·17 | 30·17 | 70 | 56 | 61·3 | Fair |
| | 31 | NW | 30·37 | 30·20 | 30·307 | 71 | 58 | 62·6 | Fair |
| | 1 | NbyW | 30·38 | 30·37 | 30·379 | 73 | 59 | 64 | Fair |
| | 2 | W | 30·32 | 30·25 | 30·282 | 74 | 58 | 64 | Fair |
| | 3 | WbyS | 30·25 | 30·25 | 30·25 | 72 | 56 | 62 | Fair |
| | 4 | W | 30·24 | 30·16 | 30·213 | 70 | 55 | 61 | Fair |
| | 5 | NW | 30·12 | 30·12 | 30·12 | 65 | 55 | 58·6 | Fair |
| ◑ | 6 | WbyN | 30·12 | 30·02 | 30·062 | 66 | 57 | 59·6 | Fair |
| | 7 | WbyN | 29·82 | 29·76 | 29·782 | 65 | 56 | 58·3 | Fair |
| | 8 | W | 30·09 | 29·95 | 30·212 | 62 | 54 | 56 | Fair |
| | 9 | NW | 30·10 | 30·09 | 30·097 | 62 | 55 | 56·6 | Fair |
| | 10 | NW | 30·12 | 30·08 | 30·105 | 69 | 54 | 59·6 | Fair |
| | 11 | W | 30·12 | 30·09 | 30·102 | 70 | 56 | 61·3 | Fair. Aurora Bor. at night. |
| | 12 | SW | 30·14 | 30·14 | 30·14 | 72 | 53 | 61·3 | Fair |
| ● | 13 | WbyN | 30·15 | 30·13 | 30·137 | 68 | 52 | 58 | Fair |
| | 14 | NW | 30·13 | 30·13 | 30·13 | 65 | 49 | 55·3 | Fair |
| | 15 | NW | 30·13 | 30·12 | 30·127 | 64 | 48 | 55 | Fair |
| | 16 | NW | 30·07 | 30·04 | 30·055 | 64 | 49 | 54·3 | Fair |
| | 17 | NW | 30·04 | 30·04 | 30·04 | 65 | 50 | 55·3 | Fair |
| | 18 | W | 30·08 | 30·06 | 30·067 | 70 | 54 | 58·6 | Fair |
| | 19 | WbyS | 30·08 | 30·08 | 30·08 | 69 | 53 | 59 | Fair |
| | 20 | SW | 30·— | 29·97 | 29·969 | 73 | 59 | 63 | Fair |
| ◐ | 21 | S | 29·83 | 29·80 | 29·812 | 75 | 57 | 63·6 | Fair |
| | 22 | S | 29·77 | 29·77 | 29·77 | 73 | 54 | 61·6 | Rain |
| | 23 | S | 29·80 | 29·75 | 29·78 | 70 | 53 | 59·6 | Rain |
| | 24 | SW | 29·72 | 29·70 | 29·706 | 68 | 56 | 58 | Fair. Rain & thund. at night |
| | 25 | SW | 29·70 | 29·70 | 29·70 | 69 | 53 | 59·3 | Rain |

RESULTS.

Mean barometrical pressure 30·051 | Mean temperature 59·8 deg.

Maximum 30·38 wind at NW | Maximum 75, wind at S

Minimum 29·70 ——— SW | Minimum 48, ——— NW

*Scale exhibiting the prevailing Winds during the Month.*

| N | NE | E | SE | S | SW | W | NW |
|---|---|---|---|---|---|---|---|
| 0 | 0 | 0 | 0 | 3 | 4 | 13 | 10 |

| | Mean barometrical pressure. | Mean temperature. |
|---|---|---|
| From the full moon on the 30th August, to the last quarter on the 6th Sept. | 30·537 | 61·9 |
| ——— last quarter, to the new moon on the 13th | 30·082 | 58·9 |
| ——— new moon, to the first quarter on the 21st | 30·022 | 57·3 |

Cases

Cases of cholera, diarrhœa, and colic, have been frequent: amongst the latter were some instances of Colica pritonum. Jaundice has been more than usually prevalent; and some distressing instances of bilious vomiting have occurred. Synochus has affected several persons, but no fatal case has happened within the reporter's observation. Scarlatina anginosa, of a mild character, is just now very prevalent: in a large ladies' school where several children are affected with it, the symptoms are so slight, that the only treatment pursued has been purgatives, and cold water as a drink, with directions to the nurse to sponge the body should the fever at any time be high.

